# Analysis of the Influence of Surface Modifications on the Fatigue Behavior of Hot Work Tool Steel Components

**DOI:** 10.3390/ma14237324

**Published:** 2021-11-30

**Authors:** Thomas Wild, Timo Platt, Dirk Biermann, Marion Merklein

**Affiliations:** 1 Institute of Manufacturing Technology (LFT), FAU Erlangen-Nürnberg, 91058 Erlangen, Germany; thomas.wild@fau.de (T.W.); marion.merklein@fau.de (M.M.); 2 Institute of Machining Technology (ISF), TU Dortmund University, 44227 Dortmund, Germany; dirk.biermann@tu-dortmund.de

**Keywords:** fatigue, surface modification, micromilling, high-feed milling, grinding, residual stresses, AISI H11

## Abstract

Hot work tool steels (HWS) are widely used for high performance components as dies and molds in hot forging processes, where extreme process-related mechanical and thermal loads limit tool life. With the functionalizing and modification of tool surfaces with tailored surfaces, a promising approach is given to provide material flow control resulting in the efficient die filling of cavities while reducing the process forces. In terms of fatigue properties, the influence of surface modifications on surface integrity is insufficiently studied. Therefore, the potential of the machining processes of high-feed milling, micromilling and grinding with regard to the implications on the fatigue strength of components made of HWS (AISI H11) hardened to 50 ± 1 HRC was investigated. For this purpose, the machined surfaces were characterized in terms of surface topography and residual stress state to determine the surface integrity. In order to analyze the resulting fatigue behavior as a result of the machining processes, a rotating bending test was performed. The fracture surfaces were investigated using fractographic analysis to define the initiation area and to identify the source of failure. The investigations showed a significant influence of the machining-induced surface integrity and, in particular, the induced residual stress state on the fatigue properties of components made of HWS.

## 1. Introduction

The current challenges regarding the ecological and economic manufacturing of components require constant technical improvement of existing processes. In particular, lightweight construction, functional integration and resource efficiency are essential aspects that define the competitiveness of modern manufacturing technologies [1]. By means of bulk forming and especially extrusion, a suitable method is provided for the economical large-scale production of complex components. Further advantages of this process class are high material utilization and load-adapted hardening of the components due to the plastic forming [2]. However, the high process forces of bulk forming in combination with complex component geometries with sharp radii result in high local tool loads [3]. Therefore, tool life may be limited to approximately 10^3^–10^4^ produced parts [4]. For those high-performance tools such as dies, which are often made of hot work tool steels with high levels of strength, hardness and toughness [5], considerable effort is dedicated to the surface finishing in order to reduce friction and to improve the forming quality. This can be provided by polishing or coating the tool surfaces to reduce the resulting surface roughness [6], which can also affect the resulting fatigue behavior. Fatigue properties in general are related to crack propagation, which often initiates from persistent slip bands [7], grain boundaries [8] or surface irregularities such as scratches in the material’s surface [9], implicating the impact of the surface topography on the fatigue behavior. Rough surfaces can lead to critical stress intensification and premature tool failure due to the notch effect [10]. In addition, material properties such as microstructure, microhardness and the residual stress state can significantly affect the fatigue strength of materials. Characteristics of the near-surface residual stress state have a significant effect on the fatigue strength of metallic materials [11]. In particular, compressive residual stresses have the potential to reduce the propagation of cracks and surface defects [12].

In the evaluation of the predominant factors regarding the influence of surface roughness, the residual stress state or the hardness on the fatigue strength, various machining processes have been studied in detail with regard to surface integrity, observing correlations for the specific machining processes. Investigations on the fatigue strength of hard-turned specimens revealed that a higher residual compressive stress can compensate for a higher surface roughness [13]. However, in the case of shot peening, a differing dependency for these influencing factors was found. Despite the significant compressive residual stress state as a consequence of this surface treatment, a critical value of surface roughness was identified, causing the fatigue strength to decrease above this value [14]. Comparing the influence of milling strategies on a high alloy steel, X160CrMoV12, regarding the fatigue strength showed that detrimental roughness effects are less relevant than the benefits of surface hardening and high compressive stresses [15]. In a study of high-speed milling of AISI H13, different tool path orientations resulted in varying values of effective residual stress, which affected the fatigue behavior of the workpiece [16]. A further essential factor involves the depth distribution of the compressive residual stress state. Depending on the parameter selection while conducting the shot peening, an increased depth distribution of the compressive residual stress state provides a higher fatigue strength [17].

In this work, these cause–effect relationships were investigated within the field of tool systems in sheet bulk metal forming. This process class is characterized by the high tool loads that occur due to the forming of filigree functional elements [18] and high contact pressures that result in increased tool wear [19]. In order to improve the process results in this field, the application of surface structuring methods, so-called tailored surfaces, were used to control the material flow during forming [20]. Since a large portion of the cost per produced part is influenced by the cost of tool fabrication [21], accurate knowledge of the impact of surface modifications on the fatigue behavior of hot work tool steel components is essential to design efficient production processes. The reference surface treatment for the investigations within this work was abrasive grinding to achieve a fine surface structure, as this procedure represents an industrial standard. Furthermore, in cases of appropriate selection of the grinding process parameters and avoidance of thermal influence on the surface, a compressive residual stress state was introduced into the ground surface [22]. For modification of the surfaces, the high-feed milling and micromilling machining processes were selected. While the potential of the mentioned processes in the context of tailored surfaces and, thus, the functionalization of the surface topography of forming tools has been demonstrated in various publications [23], the resulting effects on fatigue properties are poorly studied. Therefore, specimens were modified and tested in the rotating bending test to evaluate the effect of surface properties on fatigue behavior. Since the resistance of the specimens to stress depends on the surface integrity, the surface properties as well as the residual stress state, these parameters were analyzed and related to the fatigue behavior. Influencing factors regarding fracture behavior were also derived to generate a holistic view of process-dependent surface properties. 

## 2. Materials and Methods

**Figure 1:**
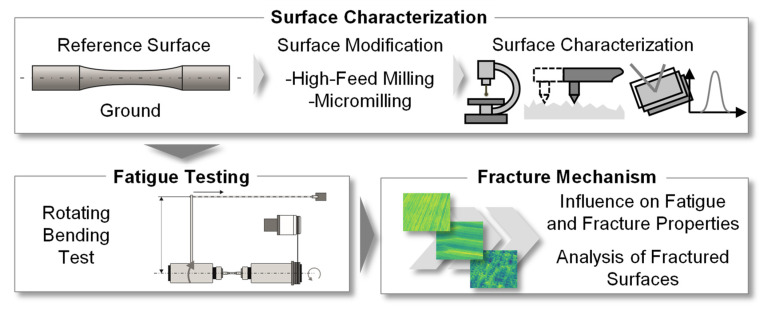
Methodical approach of the investigation.

The objective of this work was to analyze the effect of surface modifications on the fracture mechanisms and the fatigue strength of hot work tool steel samples. Thereby, process knowledge regarding the industrial applicability of so-called tailored surfaces needed to be generated, since these surface modifications proved to be promising in, e.g., forging processes for controlling the material flow [20]. The functionality and applicability of these modified surfaces is strongly dependent on the machining process-related properties in terms of topography design, residual stress state and fatigue behavior. For this reason, surface-modified specimens were tested in a laboratory test according to the methodical approach in Figure 1. For this work, the reference surface was a ground finish, as this represents a widespread procedure in tool manufacturing. Based on this reference, the hourglass-shaped specimens were modified by high-feed milling and micromilling, in order to generate a modified surface condition in terms of roughness/surface topography and residual stress. 

To derive cause–effect relationships between the surface properties and the resulting fatigue strength, a comprehensive analysis of the specimens was performed as a first step. This was followed by the conducting of the fatigue tests and the calculation of fracture probabilities by means of the arcsin√P- transformation [24]. Subsequent microscopic analysis of the fracture surfaces and edges was used to generate further understanding of the surface influence on fracture behavior.

### 2.1. Material and Surface Modification

**Figure 2:**
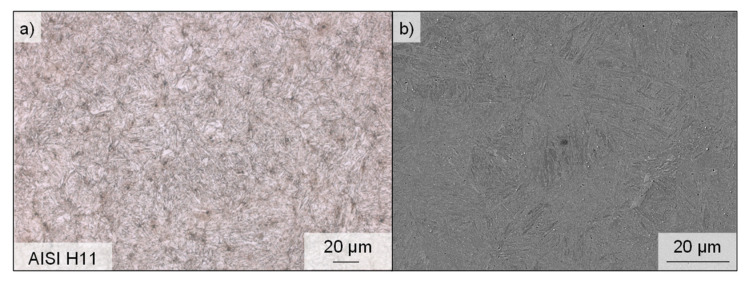
(**a**) Optical and (**b**) SEM micrographs of the microstructure of AISI H11 hot work tool steel.

For the investigation of the process-related potential of surface modifications with regard to the implications for the resulting fatigue properties, specimens of HWS (AISI H11), hardened to approximately 50 ± 1 HRC, were used. To verify the hardening, each specimen was tested at three locations using a hardness testor (Instron Wolpert, Testor 930, Ludwigsburg, Germany). As a result of these measurements, 49.71 ± 1.59 HRC was determined. This material is also characterized approximately by a tensile strength R_m_ = 1800 MPa and yield strength R_p0.2_ = 1460 MPa at room temperature [25]. Figure 2 shows the microstructure consisting of acicular, tempered martensite. The specimen was polished and etched slightly with a 3% Nitric acid (HNO_3_) in alcohol (nital). Subsequently, micrographs were produced using a digital light microscope and a secondary electron microscope (SEM) (Tescan, Mira III XMU, Dortmund, Germany). Due to the high toughness and hot strength of this tool steel, as well as its low sensitivity to hot cracking and good thermal conductivity, the high-alloy tool steel is one of the most widely used materials for tools operating under high thermal and mechanical stress in applications such as hot forging, extrusion and die casting. The high ductility makes this material also well-suited for use in critical aircraft parts such as helicopter rotors, landing gear components [26] or airframe structures [27]. The composition of alloying elements in AISI H11 is C = 0.36, Si = 1.00, Mn = 0.3, Cr = 5.00, Mo = 1.3, V = 0.4 (values specified in wt%) [28].

**Figure 3:**
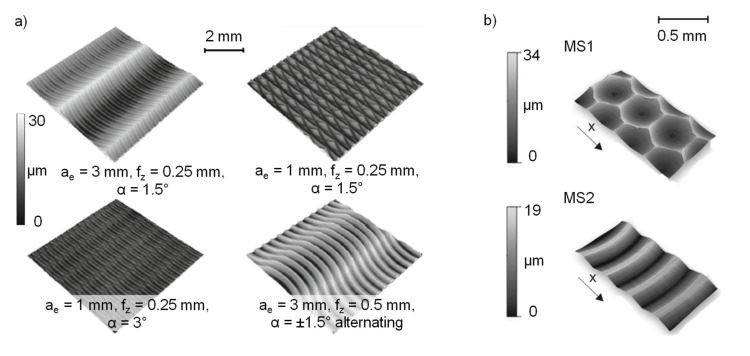
Surface structures (**a**) high-feed milling [31] and (**b**) micromilling [32].

As described below, various machining processes were used to produce different surface finishes. The high-feed milling was conducted on a 5-axis CNC machining center (DMG MORI AG, HSC 75 linear, Bielefeld, Germany). A cutting speed *v*_c_ = 50 m/s and a lead angle of 3° were used with a cutting tool geometry containing a typical small cutting edge angle, leading to less radial and higher axial forces. High-feed milling in general is a machining process that is used to realize high material removal rates in the rough machining of hardened materials [29]. Compared to the conventional milling process, high feeds per tooth with a low axial cutting depth are used [30]. Depending on the cutting edge geometry of high-feed mills and the process parameter values for the feed per tooth f_z_, the width of cut a_e_ and the lead angle α, a wide range of functional surfaces can be applied with quasi-deterministic surface structures, see Figure 3. Therefore, high-feed milling is suitable for controlling surface properties, e.g., roughness and residual stress states on complex and especially on large surface areas [23]. 

The micromilled specimens were prepared on the machine tool (KERN Microtechnik, HSPC 2522, Eschenlohe, Germany), which is highly suitable for micromachining tasks due to its high working accuracy of 2.5 μm and the rotational speed range of the tool spindle (VSC 4084 Precise) of up to *n* = 50,000 rpm. The acceleration capabilities of the machine tool are specified as *a*_max_ = 2000 mm/s^2^ and a maximum feed rate of *v*_f,max_ = 6000 mm/min is provided. The surface modification was conducted with two-fluted micro ball nose end mills with a diameter of *d* = 1 mm. The substrate consisting of ultra-fine grain cemented carbide (WC grain size 0.2–0.5 μm) and the applied TiAlN PVD-coating qualify these tools for the hard machining of tool steels. Due to its high flexibility and accuracy, micromilling is a suitable process for the machining of small functional elements in difficult-to-access areas of dies and molds made of hardened tool steels [33]. The possibility of micromachining individual structural elements allows surfaces to be modified with respect to the topography-dependent friction as well as the residual stress state [34].

### 2.2. Fatigue Life Testing

**Figure 4:**
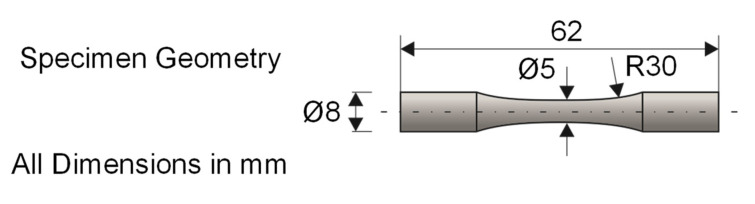
Test setup and specimen geometry of the rotating bending test.

The rotating bending test, in line with DIN 50113 [35], was conducted on a testing machine (Zwick/Roell, UBM 200 tC, Ulm, Germany) for the experimental determination of the fatigue strength of the hot work tool steel H11. In Figure 4, a depiction of the used hourglass-shaped specimen geometry is given. These were fixed in the testing machine by a mechanical clamping device and rotated by a drive spindle with a rotational speed of *n* = 6000 rpm at room temperature until fracture. By means of a defined test weight attached to a lever arm, the test load was applied to the specimen. The accuracy of the used weights complie with the OIML standard M1. Due to the bearing according to the principle of four-point bending, a constant bending moment, with its maximum in the test area of the specimens (*d* = 5 mm), was achieved. As a consequence of the superposition of rotation and the simultaneous bending of the specimen, cyclic and sinusoidal loading with a near-surface bending stress maximum occurred. This context implied the particular suitability of this laboratory experiment for the evaluation of the surface-modified specimens. During testing, the objective was to select two load levels that result in fracture cycles *N* representing high cycle fatigue [36]; thus, *N* = 10^4^ and *N* = 2 × 10^6^ were selected as lower and upper boundaries for the experiments. To identify the load levels for the experiments, the procedure by DIXON and MOOD [37] was applied. If a load level did not meet the fracture cycle boundaries, the load was adjusted accordingly in steps of 150 MPa. On both load levels, five repetitions of the experiments were carried out in order to obtain a data basis for the statistical analysis. This was carried out via the calculation of curves for the fracture probabilities for each experimental series using the arcsin√P- transformation according to Dengel [24]. This procedure was initiated with the calculation of the fracture probabilities of each specimen. Subsequently, by means of a derived transformation variable and via the regression of coefficients, a best fit line was generated for the calculation of load cycles for the desired fracture probabilities. In this study, the curves with fracture probabilities of 10%, 50% and 90% of each specimen surface condition are discussed to reflect the variance in fatigue life testing.

### 2.3. Surface Characterization and Fractography

In order to analyze the relationships between the surface properties and the fracture mechanisms, surface characterizations were carried out in advance of fatigue life testing and subsequent fractographic investigations. In the first step, topography images were produced with a confocal laser-scanning microscope (Keyence, VK-X200, Neu-Isenburg, Germany) to obtain a qualitative overview of the generated surface structures. Following this, roughness values for each specimen were determined using a Perthometer (Mahr, MarSurf GD120, Göttingen, Germany) measuring five roughness profiles, respectively. To evaluate the surfaces, the industrially common parameters for the arithmetic mean roughness *Ra* as well as the reduced valley depth *Rvk* were used. The latter provides information about the score marks on the machined surfaces [38] and, thus, is suitable for discussing the chosen surface modification methods. In addition, an important parameter that influences the fatigue behavior of metals is the residual stress state [17]. Therefore, X-ray diffraction measurements (Seifert, XRD 3003, Ahrensburg, Germany) were performed. This method allows the calculation of the residual stress state based on the diffraction and reflection of monochromatic X-rays on a three-dimensional lattice structure. Reflection of the X-rays occurs at a high intensity when the Bragg condition [39] is fulfilled and the reflected X-rays interfere constructively [40]. By measuring the intensity of the reflection and determination of the Bragg angle θ of a distorted grid and comparing it to an undistorted one (intensity peak for α-Fe at 2θ = 156.084° [41]), the residual stress calculation is possible. For this evaluation the sin^2^Ψ method [40] was used. The measurements were performed on a Seifert XRD 3003 using the *χ*-method [42]. A CrKα-anode, operating at 40 kV and 40 mA, was used as a radiation source, which generated a beam diameter of 1 mm with the usage of a collimator and lens setup. The 2θ range of 146° to 164° was selected with a resolution of 0.05°-steps and an exposure time of 10 s for the measurements. The Ψ-tilt of the samples was performed in a range of ±40°, which was divided into 13 steps based on the sin^2^-distribution. Vickers microhardness tests were performed according to Din EN ISO 14577-1 using a nanoindentation tester (Shimadzu Corp., DUH-211S, Kyoto, Japan) with a load of 0.1 g (HV0.1). For evaluation of the fractured surfaces and for fractographic analysis, an optical focus variation microscope (Bruker Alicona, Infinite Focus G5, Graz, Austria) was used to digitize the samples to define the initiation area and identify the source of failure. In addition, the fracture surfaces were measured with a SEM (Tescan, Mira III XMU, Dortmund, Germany) to obtain detailed information about the features or fatigue damage.

## 3. Results and Discussion

As the surface integrity strongly affects the fatigue behavior of metals [13], the results of the surface characterization, the fatigue testing and the subsequent fractographic analysis of surface-modified specimens made of HWS AISI H11 are discussed in the following section. This process is intended to generate an overall understanding of the influence of surface processing on fracture mechanisms of the investigated hot work tool steel.

### 3.1. Surface Topography and Residual Stress State

**Figure 5:**
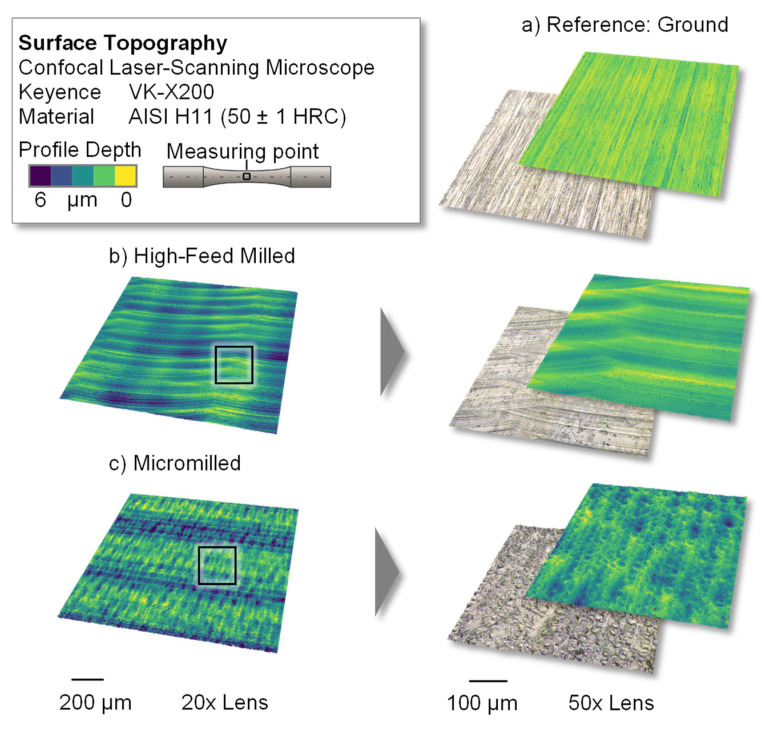
Resulting surface topographies of the (**a**) ground, (**b**) high-feed milled and (**c**) micromilled specimens.

To provide an overview of the resulting surfaces, topography images of the ground, high-feed milled and micromilled surfaces were produced with a confocal laser-scanning microscope and are presented in Figure 5. In the ground reference version (Figure 5a), characteristic grooves, which occurred due to the processing with abrasive particles, are visible. These display a low profile depth and are arranged at regular distances. The depicted surface topography of the high-feed milled specimens (Figure 5b) shows a quasi-deterministic surface structure that resulted from the combination of the values for the lead angle, the feed per tooth and the width of the cut with the special geometry of the high-feed milling tool, which had adjusted cutting edges.

The micromilled surface (Figure 5c) shows an irregular topography with crater-like depressions. Following the process kinematics of a mill-turn-like micromilling process, the feed direction of the used ball nose cutter followed a convex path perpendicular to the specimen axis. Since the cutting speed of the tool nose was zero, ploughing effects with relatively high passive forces could cause vibration of the workpiece during the cutting process, which could have contributed to the irregular surface topography.

**Figure 6:**
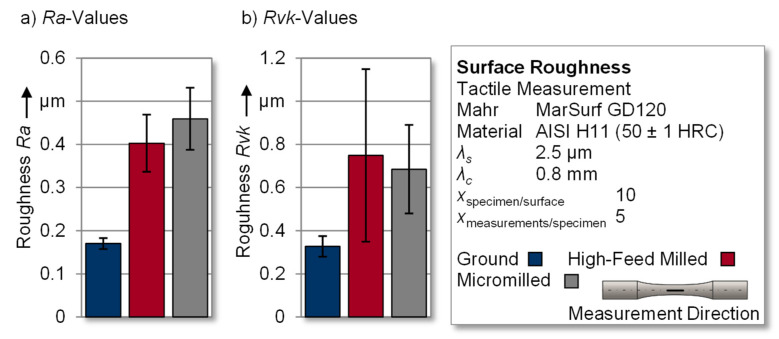
(**a**) Ra and (**b**) Rvk roughness values of the modified specimens.

The discussion of the resulting roughness values of the surface-modified specimens is carried out using the arithmetic mean roughness *Ra* (see Figure 6a) and the reduced valley depth *Rvk,* (see Figure 6b). This selection is based on the fact that the arithmetic mean roughness *Ra* represents a general overview of the surface roughness and is widely used in industry, whereas the reduced valley depth *Rvk* reflects the portion of generated grooves [38]. Therefore, the latter represents a relevant parameter for the discussion of fatigue testing results, since grooves and notches are a potential initiating point for fatigue failure [10]. For each specimen, five roughness profiles were measured in the test area to generate a qualitative overview of the investigated surfaces. Therefore, 50 individual roughness profiles were measured per surface modification and used to calculate the mean value in order to obtain valid results.

Starting with the ground reference surfaces, the specimens exhibited mean roughness values of *Ra* = 0.170 ± 0.012 µm and *Rvk* = 0.327 ± 0.048 µm, which also represented the original topography before processing by micro- and high-feed milling. Regarding the high-feed milled surface, a pronounced surface texture (see Figure 5b) was introduced to the hot work tool steel, which resulted in an *Ra* value of 0.402 ± 0.066 µm. The crater-like structure of the micromilled specimens (see Figure 5c) caused increased roughness values of *Ra* = 0.459 ± 0.072 µm. Comparing the gradation of the *Ra* roughness values with the underlying groove structure of the surfaces, a distinctive aspect was identifiable. Overall, the *Rvk* values for micromilling and high-feed milling were above the reference values of *Rvk* = 0.327 ± 0.048 µm for the ground surface. However, the maximum *Rvk* value was found in the high-feed milled specimens, at *Rvk* = 0.749 ± 0.400 µm. This indicates that micromilling in this case led to a higher general surface roughness, although the groove structure, according to the Abbot curve, was comparatively lower (*Rvk* = 0.685 ± 0.205 µm).

**Figure 7:**
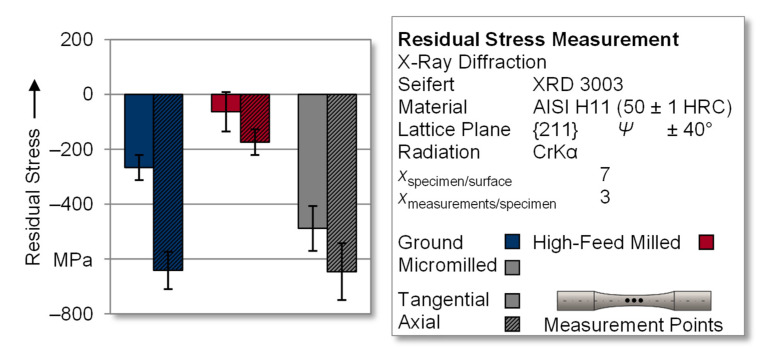
Residual stress measurements of the surface-modified specimens.

In order to enable the subsequent derivation of correlations between fatigue behavior and residual stress states of the surface-modified specimens, measurements were carried out using an X-ray diffractometer. All specimens were ground prior to the modification processes, resulting in a specific stress state. The use of a machining depth of at least 100 µm reduced the influence of the preliminary condition on the resulting residual stress state. To ensure the statistical validity of the results, seven specimens of a test series were measured at three locations within the test area. This resulted in a total of 21 individual measurements of which the mean values were calculated. These results are given in Figure 7. Grinding caused a pronounced compressive residual stress state with directionally dependent results. During fabrication of the specimens, the grinding wheel engaged radially, resulting in the removal of tangential stock. This generated higher residual compressive stresses in the axial direction (*σ_axial_* = −641.8 ± 67.8 MPa) compared to the tangential residual stresses (*σ_tangential_* = −267.0 ± 45.9 MPa). Similar effects with the formation of the residual states dependent on the grinding direction can also be found in the literature [12]. An analogous correlation between tangential and axial residual stresses was obtained as a result of the investigated surface modifications; however, the mechanical stock removal during high-feed milling led to a significantly lower residual compressive stress state. In the tangential direction, residual stress values of *σ_tangential_* = −63.0 ± 71.8 MPa were measured, and in the axial direction, residual stress values *σ_axial_* = −174.4 ± 46.6 MPa were measured. The measurements in the tangential direction showed values in the positive as well as the negative residual stress range, which led to a correspondingly high deviation range compared to the mean value in this spatial direction. Micromilling generated a distinct residual compressive stress state again. In contrast to the ground reference specimens, the directionality was not as pronounced, with values of *σ_tangential_* = −488.6 ± 81.8 MPa and *σ_axial_* = −646.5 ± 103.4 MPa for the tangential and axial directions, respectively.

While high-feed milling is generally capable of producing relatively high values for residual compressive stresses, the measured values in this study appeared to be rather low. In a similarly designed study, the identical machining process for the high-feed structuring of high-speed steel 1.3344 also showed comparable levels of residual stresses, which were slightly higher due to the higher hardness of the workpiece material [43]. This can be attributed in particular to the NC paths caused by the specimen’s geometry. The changed process kinematics when machining round specimens seems to generate lower residual stresses than for flat specimens due to the different process characteristics.

**Figure 8:**
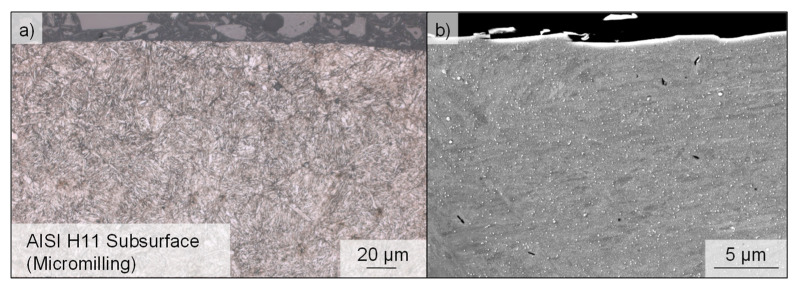
(**a**) Optical and (**b**) SEM micrographs of the near-surface microstructure of the hot work tool steel AISI H11.

Figure 8 shows SEM and optical micrographs of the near-surface microstructure of the hot work tool steel after machining by micromilling. The homogeneous microstructure shows no clear signs of a microstructural change due to thermal or mechanical effects as a result of machining.

**Figure 9:**
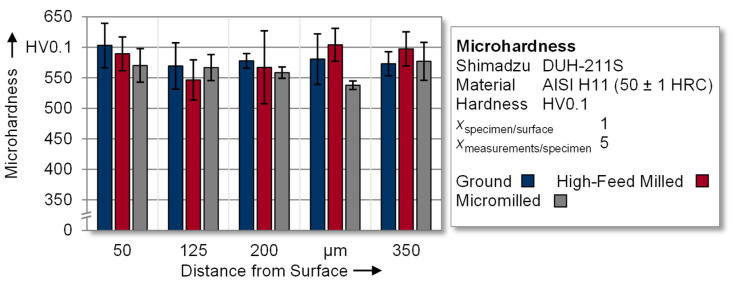
Depth distribution of the near-surface microhardness.

In Figure 9, near-surface microhardness measurements of the surface-modified specimens are evaluated in order to analyze the influence of the machining process on the depth distribution. The individual measured values in the range from 540 to 600 HV0.1 indicate that no significant and systematic influence of the machining process on the hardness distribution in the near-surface area is evident. Thus, it can be assumed that, in this study, the microhardness distribution had a subordinate influence on the fatigue behavior.

### 3.2. Fatigue Properties

**Figure 10:**
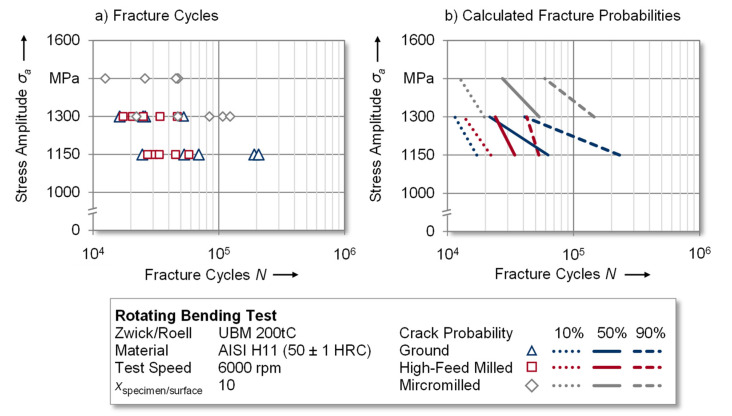
Results of the (**a**) fatigue test and (**b**) calculated fracture probability of the conducted experiments.

In order to determine the fatigue properties of the investigated surface integrities, rotating bending tests were conducted on different load levels (see Figure 10a). For the ground and high-feed milled specimens *σ_a_* = 1150 and *σ_a_* = 1300 MPa were determined to be adequate load levels according to the test methodology presented in Section 2.2. The experimental results of both surface states showed higher achievable fracture cycles with a higher variance for ground specimens in comparison to the high-feed milled ones. Micromilling enabled fatigue testing at higher load levels of *σ_a_* = 1300 MPa and *σ_a_* = 1450 MPa, nevertheless achieving similar fracture cycles. It can, therefore, be concluded that the fatigue strength improved due to the more pronounced compressive residual stress state of these specimens. Since, the findings of fatigue testingare subject to variance, the following discussion of the results is based on calculated fracture probabilities as described in Section 2.2. In order to reflect the experimental variance and to create a basis for industrial tool design, the 10%, 50% and 90% fracture probabilities of the three test series are plotted in Figure 10b.

As a reference for the experiments, specimens with a ground surface finish were tested at stress amplitudes of *σ_a_* = 1150 MPa and *σ_a_* = 1300 MPa. The lower load level resulted in approximately *N* = 62,500 and the higher load level resulted in *N* = 21,500 fracture cycles at a fracture probability of 50%. According to the explanations in Section 3.1, high-feed milling led to surface structures with increased roughness values yet lower induced compressive residual stresses in comparison to the ground reference. This was also reflected in the results of the fatigue experiments, which were conducted at the same load levels as the reference experiments. Especially at the lower load level of *σ_a_* = 1150 MPa, the fracture cycles decreased to approximately *N* = 34,000 at a fracture probability of 50%. Nevertheless, the higher load level was in a comparable range to those of the reference. Compared to these two variants, the surface modification by micromilling yielded the greatest differences regarding the fatigue performance. Despite the increased surface roughness, the more pronounced residual compressive stress allowed these specimens to be tested at higher load levels of *σ_a_* = 1300 MPa and *σ_a_* = 1450 MPa. The corresponding fracture cycles at a fracture probability of 50% were circa *N* = 53,000 and 27,000, respectively. This led to the conclusion that surface modification by micromilling and the associated surface conditions, especially the residual compressive stress state, provided an improvement of the fatigue strength in the rotating bending test.

Furthermore, the influence of the residual stress state and the anisotropy of the residual stresses of surface-modified tool steels is discussed to derive functional correlations between these parameters on the resulting fatigue strength. For this purpose, micromilling and high-feed milling are initially analyzed with regard to the axial residual stress state since a compressive stress state in this direction opposes the main fracture propagation in the specimen during testing. In this study, micromilling caused the near-surface axial compressive residual stresses to be increased by approximately 270% (*σ_axial_* = −646.5 ± 103.4 MPa) in comparison to high-feed milling (*σ_axial_* = −174.4 ± 46.6 MPa), which enabled fatigue testing on higher load levels and the achieving of higher fracture cycles. However, extending this analysis to the ground specimens, a similar axial compressive residual stress state (*σ_axial_* = −641.8 ± 67.8 MPa) resulted in lower fatigue strength in comparison to the micromilled specimens. The reason for this might be that the tangential residual compressive stress (ground: *σ_tangential_* = −267.0 ± 45.9 MPa; micromilled: *σ_tangential_* = −488.5 ± 81.8 MPa) was approximately 45% lower for these specimens. It can thus be concluded that, in addition to the absolute value of the compressive residual stresses, their directional dependency yielded a significant influence on the performance of the tool steel.

### 3.3. Fractography

This section presents the analysis of the fracture morphologies by digitization and SEM measurements of the fracture surfaces of the specimens made of HWS AISI H11. The specimens were prepared by grinding, micromilling and high-feed milling and tested in the rotating bending test with a maximum applied stress amplitude *σ_a_* of 1150–1300 MPa (grinding, high-feed milling) and 1300–1450 MPa (micromilling). All tested surfaces showed typical characteristic areas within the fatigue tests, containing a clear origin (I) and stable fatigue crack growth in the fatigue zone (II), followed by an instantaneous fracture zone due to an overload (III).

**Figure 11:**
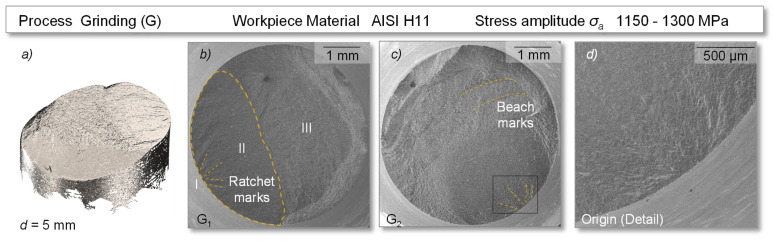
Fatigue induced fracture surface of specimens with a ground surface—(**a**) digitized fracture surface, (**b**) specimen G_1_, (**c**) specimen G_2_, (**d**) origin of cracking.

In all ground reference specimens, the crack origin could be identified by the appearance of ratchet marks, lines that ran parallel to the overall direction of crack propagation, see Figure 11. These were supplemented in half of the fracture surfaces by concentric rings, so-called beach marks, which originated from the initiation point and characteristically developed perpendicular to the overall direction of crack growth, see G_2_. Both were macroscopically visible lines that served to identify the origin and provide information about the extent of the fatigue zone and, thus, the level of nominal stress. The test ended with the instantaneous fracture zone (III). The surface topography changed qualitatively from a smooth (I) to relatively rough (III) surface.

**Figure 12:**
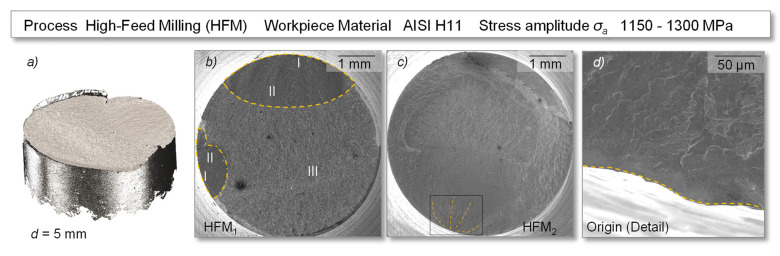
Fatigue induced fracture surface of exemplary specimens with a high-feed milled surface—(**a**) digitized fracture surface, (**b**) specimen HFM_1_, (**c**) specimen HFM_2_, (**d**) origin of cracking.

Comparable fracture surfaces were also found in half of the high-feed milled samples represented by HFM_2_ in Figure 12. The other half of the samples showed sharply defined and homogeneous fracture surfaces, without the previously mentioned beach marks, see HFM_1_. In addition, for the high-feed milled specimens, the appearance of multiple cracks could be observed in several cases. Assuming that the structurally induced reduced valley depth (*Rvk*) (see Figure 6) in the hourglass-shaped and “smooth” specimen surface could have increased the stress concentration factor, this may generally favor the occurrence of multiple origins. However, the relevance of the surface topography on the resulting fatigue life was found to be low in this study.

**Figure 13:**
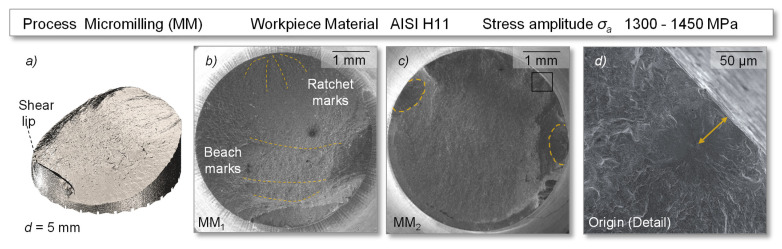
Fatigue induced fracture surface of exemplary specimens with a micromilled surface—(**a**) digitized fracture surface, (**b**) specimen MM_1_, (**c**) specimen MM_2_, (**d**) origin of cracking.

Examining the exemplary specimens MM_1_ and MM_2_, which represented the specimen size for micromilling, the fracture patterns on all specimens showed inhomogeneous surface topographies with pronounced fatigue zones (Figure 13). The origin in the crack initiation zone was complemented by the ratchet and beach marks. As seen before, fatigue crack initiation was mostly detected in concentration zones at the surface, e.g., by defects or machine-induced features. However, some fractures of the micromilled specimens, which exhibited a relatively high fracture cycle number in the tests, showed a slight displacement of the origin to the subsurface. While material inclusions may be an explanation, those would result in superimposed stress conditions that anticipate crack nucleation and generally reduce fatigue life. 

In contrast, however, a high fatigue life was measured for the specimen studied, so a more plausible reason for this could be related to the residual stress state in the surface. It can be assumed that crack initiation occurred at a distance from the surface where the applied tensile stress was approximately in the range of the compressive stress induced by micromilling [44]. In addition, multiple origins were also found on some specimens, but these showed subordinate influence on the fatigue behavior as the micromilled specimens showed high performance. Proceeding from this, an area of stable fatigue crack growth up to the fracture zone was shown. The inhomogeneous failure progression as well as the displaced origin may indicate a high fatigue strength, which supports the high number of cycles in the fatigue test compared to the ground and high-feed milled specimens.

## 4. Conclusions and Outlook

For the purpose of analyzing the influence of surface modifications by high-feed milling and micromilling on the fatigue properties of hot work tool steel components, rotating bending tests were performed and compared to ground samples. Based on the results, the following conclusions can be derived:

- The impact of the investigated surface integrities on the fatigue properties emphasizes the requirement of a considerate selection of process routes for the finishing of, e.g., highly stressed components in order to improve tool life.
- In this series of experiments, the resulting roughness and microhardness values, as a consequence of the chosen machining processes, had a minor influence on the fatigue behavior.
- In contrast, the residual stress state correlated significantly with fatigue resistance, resulting in high fatigue life due to high compressive stress states in grinding and micromilling. Thus, the surface integrity can be derived as a key specification in component design, complementing geometry and material selection. However, the adaptation requires the selection of suitable machining processes.
- Understanding machining process-dependent anisotropies in surface integrity regarding the topography or residual stress state appears to be of high relevance. Their directional interdependence with the fatigue behavior of the components may offer the potential to improve fatigue strength by adapting the process control while machining to induce a homogeneous residual stress state.

In order to generate a more pronounced understanding of the impact of surface modifications on the fatigue strength of hot work tool steel components, further research work should include different specifications of the selected modification methods and further load levels. This will enable the investigation of the interaction of surface roughness and residual stresses with respect to the fatigue strength of components. Especially in the case of depth distributions, the influence of a surface-modification-related stress state to the inner stress state and hardness distribution is an interesting aspect, which could generate fundamental knowledge of the fatigue strength of materials. Another promising approach is the subsequent coating of surface-modified tools to improve their wear resistance. In this context, it is essential to identify the extent to which this procedure affects the fatigue strength and to verify these findings by transferring the investigations to an industrial-scale forming process.

## Data Availability

Not applicable.
